# A Bayesian analysis of diagnostic timelines across Alzheimer's disease, frontotemporal dementia, and other neurodegenerative conditions

**DOI:** 10.1002/dad2.70184

**Published:** 2025-09-29

**Authors:** Ananthan Ambikairajah, David Foxe, Ann‐Marie G. de Lange, James Carrick, Sau Chi Cheung, Velandai K. Srikanth, Yun Tae Hwang, Rebekah M. Ahmed, James R. Burrell, Olivier Piguet

**Affiliations:** ^1^ Centre for Ageing Research and Translation Faculty of Health University of Canberra Canberra Australian Capital Territory Australia; ^2^ Discipline of Psychology Faculty of Health University of Canberra Canberra Australian Capital Territory Australia; ^3^ National Centre for Epidemiology and Population Health Australian National University Canberra Australian Capital Territory Australia; ^4^ School of Psychology The University of Sydney Sydney New South Wales Australia; ^5^ Brain and Mind Centre The University of Sydney Sydney New South Wales Australia; ^6^ Department of Psychology University of Oslo Oslo Norway; ^7^ Department of Psychiatry University of Oxford Oxford UK; ^8^ Royal Prince Alfred Hospital Sydney New South Wales Australia; ^9^ National Centre for Healthy Ageing Melbourne Victoria Australia; ^10^ Central Coast Clinical School University of Newcastle Newcastle New South Wales Australia; ^11^ Department of Neurology Gosford Hospital Gosford New South Wales Australia; ^12^ Concord Clinical School The University of Sydney Sydney New South Wales Australia

**Keywords:** Alzheimer's disease, diagnosis, frontotemporal dementia, neurodegeneration

## Abstract

**INTRODUCTION:**

Timely diagnosis is crucial for managing neurodegenerative conditions. This study investigated whether time from symptom onset to diagnosis differs by clinical syndrome and sex.

**METHODS:**

This retrospective, cross‐sectional study included 591 participants with Alzheimer's disease (AD), frontotemporal dementia (FTD) subtypes (behavioral variant FTD [bvFTD], semantic dementia [SD], and progressive non‐fluent aphasia), logopenic progressive aphasia (LPA), and syndromes associated with movement disorders (corticobasal syndrome, FTD with motor neuron disease [FTD‐MND], and progressive supranuclear palsy). Bayesian regression models were used to compute diagnostic timelines.

**RESULTS:**

Compared to AD (3.35 years; 95% credible interval [CrI]: 3.03–3.72), SD and bvFTD had additional delays of 9.7 (95% CrI: 1.96–20.64) and 14.82 months (95% CrI: 6.94–25.42), respectively, while FTD‐MND was shorter by 11.62 months (95% CrI: −15.7 to −4.68). Men with bvFTD had 23.64 month longer delays than women (95% CrI: 10.35–44.33).

**DISCUSSION:**

Diagnostic delays may reflect syndrome‐specific clinical features, diagnostic complexity, and sociocultural factors. Findings highlight the need for improved diagnostic pathways and pre‐clinical biomarkers to facilitate earlier identification.

**Highlights:**

- Bayesian analyses revealed that diagnostic delays differ by syndrome and sex.
- Alzheimer's disease (AD) was diagnosed on average 3.35 years after symptom onset.
- Diagnoses were delayed in semantic and behavioral variant frontotemporal dementia (bvFTD) compared to AD.
- Men with bvFTD had longer delays than women.
- Findings support need for improved diagnostic pathways and pre‐clinical biomarkers.

## 1 BACKGROUND

Global estimates indicate that approximately 69 million people currently live with dementia, and this number is projected to reach 152 million by 2050.^1^, ^2^ Early detection and accurate diagnosis are crucial for access to appropriate support services, care planning, and targeted management strategies to maximize quality of life for individuals with dementia and their families. Timely diagnosis also enables clinical trial participation and, when available, implementation of disease‐modifying therapies to treat or slow disease progression.

The etiology of dementia is heterogenous and includes Alzheimer's disease (AD) and frontotemporal dementia (FTD), which have distinctive clinical presentations and trajectories of disease progression. In AD, symptom onset is often insidious, with early deficits in episodic memory^3^, ^4^ potentially misattributed to normal aging.^5^ In contrast, behavioral variant FTD (bvFTD), the most common form of FTD, presents with distinct behavioral and personality changes, including disinhibition, apathy, loss of sympathy/empathy, and dietary changes,^6^, ^7^ with limited or absent insight into these changes.^8^ Significant variability also exists within the language presentations of dementia. Progressive non‐fluent aphasia (PNFA) is characterized by agrammatism in language production and apraxia of speech, while object knowledge and single‐word comprehension is often spared.^7^ Semantic dementia (SD), conversely, is typified clinically by word‐finding difficulties for uncommon words, with compensatory mechanisms potentially supporting comprehension during routine conversation that do not necessitate understanding every single word.^7^, ^9^ Logopenic progressive aphasia (LPA), an atypical presentation of AD, features impaired single‐word retrieval and sentence repetition with preserved object knowledge and motor speech.^7^ Further complexity is added by motor syndromes associated with FTD including corticobasal syndrome (CBS),^10^ presenting with asymmetric parkinsonism and limb apraxia; progressive supranuclear palsy (PSP), characterized by early falls and vertical gaze palsy;^11^ and FTD with motor neuron disease (FTD‐MND), in which cognitive and behavioral changes co‐occur with progressive motor weakness.^12^ Therefore, differences in diagnostic timelines across neurodegenerative conditions may reflect underlying disease mechanisms, clinical presentation patterns, and diagnostic complexity.

Considerable variability exists in diagnostic timelines across neurodegenerative syndromes, with inconsistent findings reported in the literature. Many studies have either grouped all dementia types together,^13^, ^14^, ^15^, ^16^, ^17^, ^18^, ^19^, ^20^, ^21^, ^22^, ^23^, ^24^ and/or focused on AD,^24^, ^25^, ^26^, ^27^, ^28^, ^29^, ^30^, ^31^, ^32^, ^33^ younger‐onset dementia,^26^, ^34^, ^35^, ^36^ late‐onset dementia,^26^, ^27^, ^35^, ^36^ and/or FTD^26^, ^27^, ^28^, ^33^ with limited research examining variability in diagnostic timelines between AD and FTD subtypes.^25^ This heterogeneity may, in part, contribute to the mixed findings in the literature with some studies reporting no difference between FTD and AD in time to dementia diagnosis,^25^, ^26^, ^33^ while other studies indicate a longer time to diagnosis from symptom onset for FTD, compared to AD, with estimates ranging from 4.56 months^27^ to 20.1 months.^28^ Notably, studies have consistently not detected an effect of sex on diagnostic timelines.^16^, ^25^, ^26^, ^27^, ^33^, ^35^, ^36^, ^37^ However, emerging evidence indicates sex differences in disease progression,^38^, ^39^, ^40^ which may differ across diagnostic subgroups.

Understanding variation in diagnostic timelines across neurodegenerative conditions can inform targeted resource allocation and improve quality of life for people with dementia and their families by enabling earlier access to support services and interventions. It may also reduce long‐term health‐care costs by facilitating more effective management and prolonged independent living. Despite the substantial body of literature, none of the existing studies have used Bayesian methods to account for previous estimates in their models. This is a crucial gap, as incorporating prior knowledge from existing studies and quantifying uncertainty with credible intervals, which do not depend on large sample approximations, enables more precise and robust estimates of diagnostic timelines, particularly relevant for rarer neurodegenerative conditions. Therefore, this study aims to use Bayesian methods to investigate whether the time from symptom onset to diagnosis differs by neurodegenerative conditions. Additionally, this study investigates whether diagnostic timelines within neurodegenerative conditions vary by sex.

## 2 METHODS

This retrospective, cross‐sectional study included participants from FRONTIER, the dementia research clinic in Sydney, Australia, between June 2008 and March 2025. Participants included those diagnosed with a primary neurodegenerative brain condition, including AD, frontotemporal dementia subtypes (bvFTD, SD, and PNFA), LPA, and syndromes associated with movement disorders (CBS, FTD‐MND, and PSP). Disorders such as motor neuron disease and dementia with Lewy bodies were excluded, as these are not the primary focus of FRONTIER's clinical population. Diagnoses were determined through a standardized protocol that includes comprehensive and multidisciplinary clinical assessment, cognitive examination, structural brain magnetic resonance imaging, and informant report, according to relevant clinical diagnostic criteria at the time of testing for probable bvFTD,^6^ probable “amnestic” (i.e., typical) AD,^4^ SD, PNFA, or LPA (also known as the semantic, non‐fluent, and logopenic variants of PPA, respectively),^7^ CBS,^10^ FTD‐MND,^12^ or probable PSP.^11^ In addition to relevant diagnosis, inclusion criteria consisted of available data for date of diagnosis at FRONTIER and date symptoms started (i.e., symptom onset), with symptom onset preceding date of diagnosis. Young onset was defined as symptom onset before age 65, while late onset was defined as symptom onset at or after age 65.^41^ This study follows the Bayesian Analysis Reporting Guidelines (BARG)^42^ and Strengthening the Reporting of Observational Studies in Epidemiology (STROBE) guidelines.^43^

### 2.1 Time to diagnosis

**RESEARCH IN CONTEXT**

1. **Systematic review**: Google Scholar and Elicit were used to search and identify studies examining diagnostic timelines in neurodegenerative conditions.
2. **Interpretation**: Relative to Alzheimer's disease (3.35 years; 95% credible interval [CrI]: 3.03 to 3.72 years), semantic dementia and behavioral variant frontotemporal dementia (bvFTD) had longer diagnostic timelines (i.e., from symptom onset to diagnosis) of 9.7 months (95% CrI: 1.96 to 20.64) and 14.82 months (95% CrI: 6.94 to 25.42), respectively, while FTD with motor neuron disease was shorter by 11.62 months (95% CrI: −15.7 to −4.68). Compared to women, men with bvFTD and young‐onset logopenic progressive aphasia experienced longer delays of 23.64 months (95% CrI: 10.35 to 44.33) and 20.94 months (95% CrI: 2.8 to 63.31), respectively, which may reflect differences in health care–seeking behaviors or for bvFTD, sociocultural factors influencing recognition of behavioral symptoms.
3. **Future directions**: Future research should (a) explore factors contributing to diagnosis delays across patient, carer, and health‐care systems; (b) develop pre‐clinical biomarkers to facilitate early identification; and (c) assess whether targeted educational interventions can facilitate timely and accurate diagnosis.

Symptom onset was obtained from the participant or caregiver during the initial multidisciplinary clinical assessment. The earliest diagnosis date with FRONTIER was defined as the first occurrence of a diagnosis that had met the relevant criteria for the neurodegenerative condition being investigated, provided that all diagnoses at subsequent assessments remained consistent. In cases in which clinical features evolved over time such that a diagnosis was later revised, the date of the first diagnosis that met the relevant criteria was used. Time to diagnosis was computed as the difference between the date of diagnosis and the date of symptom onset, using months and years. If month of reported symptom onset was unknown (Table 1), the onset month was imputed to July of the reported year. This approach limits potential recall error to a maximum of 6 months under the assumption that symptom onset should be uniformly distributed across the year.

**TABLE 1:** Demographic characteristics of participants.

	Young onset					Late onset					Whole cohort				
Diagnosis group	Sample size	Female (%)	Age at symptom onset (years); median [Q1, Q3]	Time to diagnosis (years); median [Q1, Q3]	Symptom onset month unknown (%)	Sample size	Female (%)	Age at symptom onset (years); median [Q1, Q3]	Time to diagnosis (years); median [Q1, Q3]	Symptom onset month unknown (%)	Sample size	Female (%)	Age at symptom onset (years); mean (standard deviation)	Time to diagnosis (years); median [Q1, Q3]	Symptom onset month unknown (%)
AD	87	40 (45.98)	57.33 [54.08, 61.21]	3 [2.12, 4.33]	40 (45.98)	38	15 (39.47)	72.25 [68.79, 74.67]	2.79 [1.92, 4.19]	14 (36.84)	125	55 (44)	61.51 (8.2)	3 [2, 4.25]	54 (43.2)
LPA	27	13 (48.15)	58.92 [52.96, 61.83]	2.92 [1.79, 4.42]	16 (59.26)	28	19 (67.86)	70.71 [67.5, 72.58]	2.29 [1.85, 3.81]	14 (50)	55	32 (58.18)	63.85 (7.78)	2.58 [1.79, 4.21]	30 (54.55)
bvFTD	124	40 (32.26)	56.58 [53.33, 60.6]	4 [2.23, 6.29]	85 (68.55)	39	13 (33.33)	70 [66.29, 72.75]	3.25 [1.96, 5.33]	21 (53.85)	163	53 (32.52)	59.2 (8.2)	3.67 [2.08, 5.96]	106 (65.03)
PNFA	31	14 (45.16)	57.67 [54.04, 60.71]	3 [2.17, 4.17]	17 (54.84)	30	15 (50)	72.58 [68.56, 75.6]	2.62 [1.6, 4.5]	12 (40)	61	29 (47.54)	64.81 (9.34)	3 [1.67, 4.5]	29 (47.54)
SD	60	28 (46.67)	56.92 [53.83, 61.4]	3.96 [2.67, 5.25]	40 (66.67)	24	12 (50)	68.88 [66.4, 70.02]	3.17 [2.19, 4.35]	15 (62.5)	84	40 (47.62)	60.52 (7.27)	3.83 [2.65, 5.04]	55 (65.48)
CBS	23	14 (60.87)	57.08 [55.12, 59.42]	3.92 [2.79, 5.67]	10 (43.48)	23	13 (56.52)	69 [66.75, 71.92]	3 [1.92, 4.42]	9 (39.13)	46	27 (58.7)	63.45 (7.2)	3.5 [2.35, 4.83]	19 (41.3)
FTD‐MND	23	7 (30.43)	57.92 [55.21, 62.42]	2 [1.38, 2.83]	11 (47.83)	11	2 (18.18)	70.5 [66.71, 72.92]	2.58 [1.62, 3.33]	8 (72.73)	34	9 (26.47)	61.65 (8.26)	2 [1.52, 3.06]	19 (55.88)
PSP	13	6 (46.15)	61.08 [57.92, 63.08]	3 [2.25, 3.42]	5 (38.46)	10	5 (50)	69.79 [67.23, 71.94]	3 [2.15, 3.25]	6 (60)	23	11 (47.83)	64.58 (5.73)	3 [2.12, 3.38]	11 (47.83)
Total	388	162 (41.75)	57.25 [53.92, 61.33]	3.33 [2.08, 5]	224 (57.73)	203	94 (46.31)	70.08 [67.25, 73.33]	2.92 [1.83, 4.42]	99 (48.77)	591	256 (43.32)	61.57 (8.21)	3.25 [2, 4.79]	323 (54.65)

Abbreviations: AD, Alzheimer's disease; bvFTD, behavioral variant frontotemporal dementia; CBS, corticobasal syndrome; FTD‐MND, frontotemporal dementia with motor neuron disease; LPA, logopenic progressive aphasia; N, number; PNFA, progressive non‐fluent aphasia; PSP, progressive supranuclear palsy; SD, semantic dementia; Q, quartile.

### 2.2 Statistical methods

All statistical analyses were conducted using R version 4.5.1 (2025‐06‐13) in Rstudio (version 2025.5.1.513). Descriptive statistics were used to summarize the demographic characteristics of the sample. Data were visualized using box and violin plots to illustrate the distribution of diagnostic timelines across groups, using ggplot2 package (version 3.5.2).

Bayesian regression models were used to quantify the association between the dependent variable, time to diagnosis from date of symptom onset, and the independent variable, neurodegenerative condition (i.e., diagnostic group). The brms package (version 2.22.0) was used to fit the model:

$$\mathrm{TimeToDiagnosi}s_{i}∼\mathrm{Gamma}\left(\alpha ,\theta_{i}\right)\mathrm{where}\;\theta_{i}=\frac{\mu_{i}}{\alpha }$$

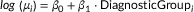

where $\mu_{i}$ is the mean time to diagnosis for individual i modelled on the log scale and α is the shape parameter, which accounts for the dispersion in the data. $\beta_{0}$ represents the intercept (log‐transformed mean time to diagnosis for the reference group, AD) and $\beta_{1}$ represents the fixed effect of neurodegenerative condition on time to diagnosis from the date of first reported symptoms. A gamma distribution with a log link function was used to model the time to diagnosis, as it is a continuous, positive outcome variable with positive‐skew.

The prior for the intercept was defined as $\beta_{0}∼N\left(log(36),0.2\right)$, which specifies a log mean time to diagnosis for AD of 36 months, with a standard deviation of 0.2 in the log space.^24^, ^25^, ^26^, ^27^, ^28^, ^29^, ^30^, ^31^, ^32^, ^44^ The prior for the fixed effect was defined as $\beta_{1}∼N\left(log(1),0.5\right)$, centered at no effect but allowing for variation across diagnostic groups.^13^, ^14^, ^15^, ^16^, ^17^, ^18^, ^19^, ^20^, ^21^, ^22^, ^23^, ^24^, ^25^, ^26^, ^27^, ^28^, ^33^, ^34^, ^35^, ^36^, ^37^, ^44^ A shape parameter of α∼N(3,0.5) was specified based on previous research.^13^, ^14^, ^15^, ^16^, ^17^, ^18^, ^19^, ^20^, ^21^, ^22^, ^23^, ^24^, ^25^, ^26^, ^27^, ^28^, ^29^, ^30^, ^31^, ^32^, ^33^, ^34^, ^35^, ^36^, ^37^, ^44^

Within diagnostic group analyses were conducted to determine whether diagnostic timelines differ between male and female participants. The prior for the intercept was defined as $\beta_{0}∼N\left(log(36),0.2\right)$, which specifies a log mean time to diagnosis for women of 36 months, with a standard deviation of 0.2 in the log space.^13^, ^14^, ^15^, ^16^, ^17^, ^18^, ^19^, ^20^, ^21^, ^22^, ^23^, ^24^, ^25^, ^26^, ^27^, ^28^, ^29^, ^30^, ^31^, ^32^, ^33^, ^34^, ^35^, ^36^, ^37^, ^44^ The prior for the fixed effect was defined as $\beta_{1}∼N\left(log(1),0.5\right)$, centered at no difference between men and women, based on previous research.^16^, ^25^, ^26^, ^27^, ^33^, ^35^, ^36^, ^37^ A shape parameter of α∼N(3,0.5) was specified based on previous research.^13^, ^14^, ^15^, ^16^, ^17^, ^18^, ^19^, ^20^, ^21^, ^22^, ^23^, ^24^, ^25^, ^26^, ^27^, ^28^, ^29^, ^30^, ^31^, ^32^, ^33^, ^34^, ^35^, ^36^, ^37^, ^44^

All analyses were repeated using young‐onset and late‐onset categories to examine whether diagnostic timelines vary based on onset. Both the diagnostic group comparisons and within‐group sex analyses used the same prior specifications as described above,^26^, ^27^, ^34^, ^35^, ^36^ with the intercept representing late‐onset AD for diagnostic group comparisons and women for sex analyses, respectively.

Sensitivity analyses were conducted and all models were re‐estimated using an alternative prior for the intercept defined as $\beta_{0}∼N\left(log(24),0.2\right)$ corresponding to a mean time to diagnosis of 24 months, with a standard deviation of 0.2 in the log space. This prior reflects a reasonable range of diagnostic timelines reported in the literature^13^, ^14^, ^15^, ^16^, ^17^, ^18^, ^19^, ^20^, ^21^, ^22^, ^23^, ^24^, ^25^, ^26^, ^27^, ^28^, ^29^, ^30^, ^31^, ^32^, ^33^, ^34^, ^35^, ^36^, ^37^, ^44^ and was used to assess the robustness of posterior estimates from prior specification.

Prior predictive checks were performed using 200 draws to assess the plausibility of priors. The models were estimated using Markov chain Monte Carlo (MCMC) sampling with eight chains, each with 8000 iterations, including 1000 warm‐up iterations, to ensure adequate sampling from the posterior distribution. MCMC convergence was assessed with $\hat{R}$ and visual inspection of trace plots. Effective sample size (ESS) was calculated for all parameters. To evaluate the model's predictive performance, leave‐one‐out cross‐validation was conducted. Posterior predictive checks were performed using 200 draws to assess the model's fit to the observed data.

To obtain the predicted time to diagnosis in months, the posterior distribution of the intercept was exponentiated. For diagnostic group comparisons, this represented the predicted time for AD; for sex analyses, this represented the predicted time for women within each diagnostic group; and for young‐onset/late‐onset analyses, this represented the predicted time for the reference category (late‐onset AD for diagnostic group comparisons, women for sex analyses). The posterior distributions of the coefficients were exponentiated to reflect the multiplicative effect on expected time to diagnosis compared to the respective reference group. A coefficient greater than 1 indicates a longer time to diagnosis, while a coefficient less than 1 indicates a shorter time to diagnosis relative to the reference group. These posterior estimates were then converted to months by multiplying the exponentiated coefficient by the exponentiated intercept. The 95% credible interval was calculated from the posterior distribution to assess the uncertainty around these estimates. Differences were considered meaningful based on the effect size and if the 95% credible interval for the exponentiated coefficient (ratio) did not include 1.0.

## 3 RESULTS

**FIGURE 1:**
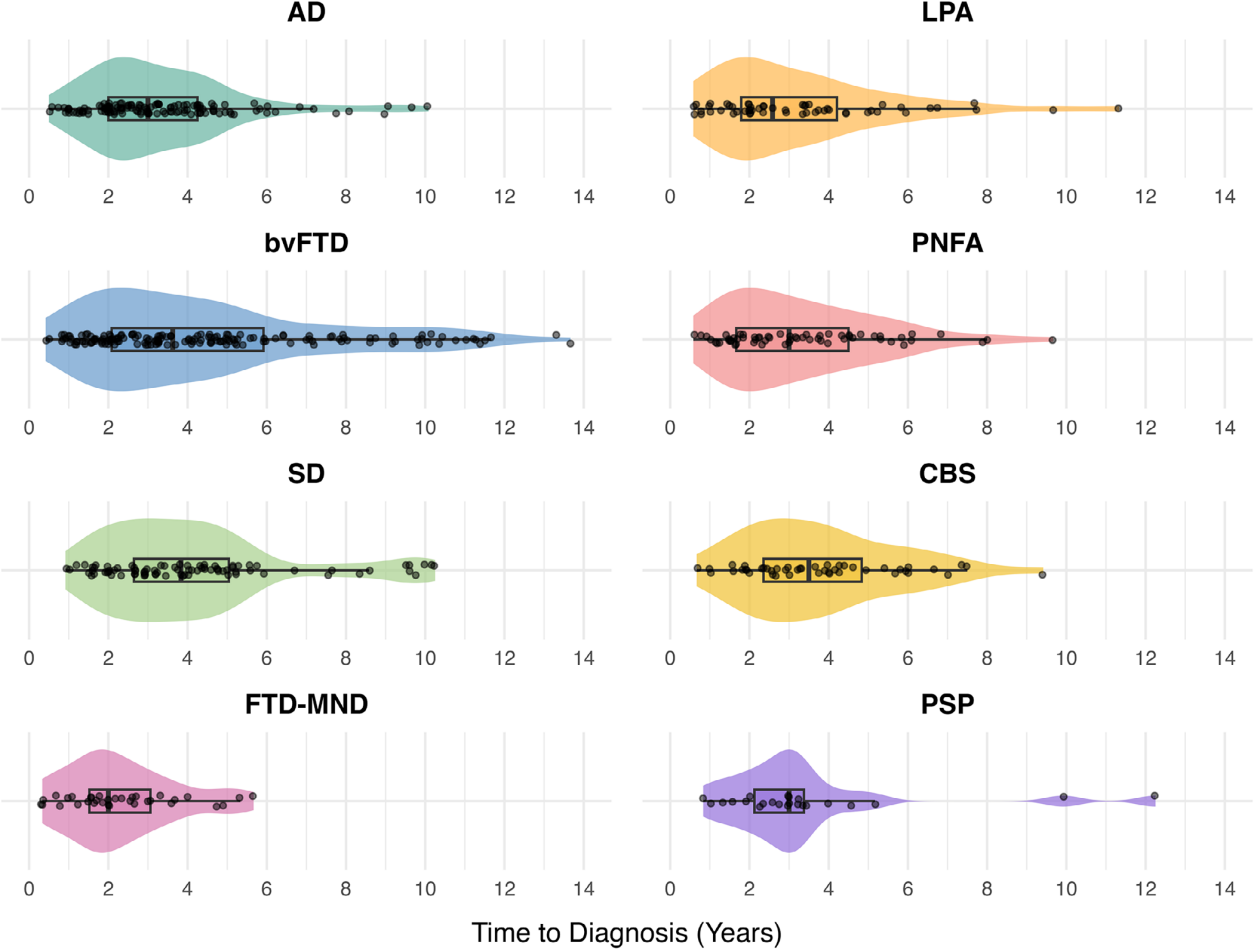
Box and violin plot of the years to diagnosis from symptom onset by disease group. Plot truncated at 14 years with 1 bvFTD participant (20.5 years to diagnosis) excluded from visualization. AD, Alzheimer's disease; bvFTD, behavioral variant frontotemporal dementia; CBS, corticobasal syndrome; FTD‐MND, frontotemporal dementia with motor neuron disease; LPA, logopenic progressive aphasia; PNFA, progressive non‐fluent aphasia; PSP, progressive supranuclear palsy; SD, semantic dementia.

Five hundred ninety‐one individuals were included in this study (AD = 125, LPA = 55, bvFTD = 163, PNFA = 61, SD = 84, CBS = 46, FTD‐MND = 34, and PSP = 23). The participants’ demographic characteristics are summarized in Table 1. The data from Table 1 indicate that typically, participants obtained a diagnosis from FRONTIER, the dementia research clinic, that meets clinical diagnostic criteria between 2 and 4.79 years from the date of symptom onset. A combined boxplot and violin plot of the years to diagnosis from symptom onset by diagnostic group is shown in Figure 1.

## 4 MODEL PERFORMANCE AND DIAGNOSTICS

Prior predictive checks confirmed that the specified priors generated plausible diagnostic timelines consistent with previous research and domain knowledge (Figures S1–S5 in supporting information). All models converged successfully, with $\hat{R}$ values < 1.01; had high resolution for reliable posterior estimation as evidenced by the effective sample size (Tables S1–S8 in supporting information); and trace plots demonstrated good mixing indicating that the MCMC chains explored the parameter space effectively. Posterior predictive checks showed that the models provided a good fit to the observed data (Figures S1–S5). Leave‐one‐out cross‐validation yielded reliable estimates (all Pareto *k* < 0.7) and supported the relative predictive performance of the final models.

### 4.1 Diagnostic group

**FIGURE 2:**
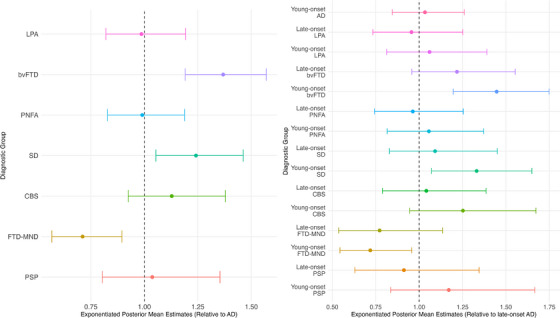
Bayesian model estimates with 95% credible intervals. On the left, estimates represent differences in time to obtaining a diagnosis from symptom onset relative to AD (reference group). On the right, estimates represent differences in time to obtaining a diagnosis from symptom onset relative to late‐onset AD (reference group). AD, Alzheimer's disease; bvFTD, behavioral variant frontotemporal dementia; CBS, corticobasal syndrome; FTD‐MND, frontotemporal dementia with motor neuron disease; LPA, logopenic progressive aphasia; PNFA, progressive non‐fluent aphasia; PSP, progressive supranuclear palsy; SD, semantic dementia.

Those with AD on average obtained a diagnosis from the date of symptom onset in 3.35 years (95% credible interval [CrI]: 3.03 to 3.72 years; Table S1). Compared to AD, individuals with SD obtained a diagnosis from the date of symptom onset 1.24 (95% CrI: 1.05 to 1.46) times later (Figure 2; Table S1), which equates to a delay of 9.7 months (95% CrI: 1.96 to 20.64 months). Similarly, participants with bvFTD obtained a diagnosis from the date of symptom onset 1.37 (95% CrI: 1.19 to 1.57) times later than those with AD (Figure 2; Table S1), which equates to a delay of 14.82 months (95% CrI: 6.94 to 25.42 months). In contrast, participants with FTD‐MND obtained a diagnosis from the date of symptom onset 0.71 (95% CrI: 0.57 to 0.9) times sooner than those with AD (Figure 2; Table S1), which equates to a diagnosis being established 11.62 months sooner (95% CrI:−15.7 to −4.68 months). No differences were observed in time to diagnosis from symptom onset between participants with LPA, PNFA, CBS, and PSP compared to those with AD (Figure 2; Table S1).

### 4.2 Onset type

Compared to late‐onset AD, individuals with young‐onset SD obtained a diagnosis from the date of symptom onset 1.33 (95% CrI: 1.07 to 1.65) times later (Figure 2; Table S2), which equates to a delay of 13.08 months (95% CrI: 2.42 to 30.09 months). Similarly, individuals with young‐onset bvFTD obtained a diagnosis from the date of symptom onset 1.45 (95% CrI: 1.2 to 1.75) times later than those with late‐onset AD (Figure 2; Table S2), which equates to a delay of 17.64 months (95% CrI: 6.64 to 34.69 months). Participants with young‐onset FTD‐MND obtained a diagnosis from the date of symptom onset 0.72 (95% CrI: 0.54 to 0.96) times sooner than those with late‐onset AD (Figure 2; Table S2), which equates to a diagnosis being established 11.1 months sooner (95% CrI: −15.41 to −1.96 months). No differences were observed in time to diagnosis from symptom onset between all other groups, compared to those with late‐onset AD (Figure 2; Table S2).

### 4.3 Sex

**FIGURE 3:**
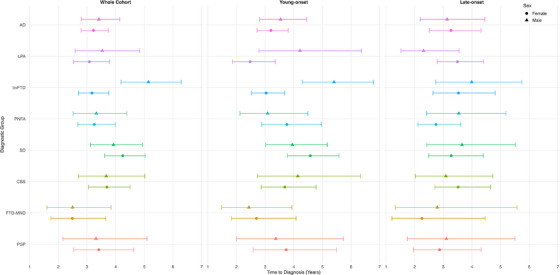
Bayesian model estimates with 95% credible intervals. Estimates represent within group analysis of sex differences in time to obtain a diagnosis from symptom onset. AD, Alzheimer's disease; bvFTD, behavioral variant frontotemporal dementia; CBS, corticobasal syndrome; FTD‐MND, frontotemporal dementia with motor neuron disease; LPA, logopenic progressive aphasia; PNFA, progressive non‐fluent aphasia; PSP, progressive supranuclear palsy; SD, semantic dementia.

Within‐group sex analysis revealed no differences in diagnostic timelines between men and women across diagnostic groups, except for bvFTD, for which men with bvFTD obtained a diagnosis from the date of symptom onset 1.62 (95% CrI: 1.32 to 1.98) times later than women with bvFTD (Figure 3; Table S3). This equates to a delay of 23.64 months (95% CrI: 10.35 to 44.33 months). These findings were isolated within young‐onset bvFTD, with men obtaining a diagnosis 1.78 (95% CrI: 1.41 to 2.24) times longer than women from symptom onset (Figure 3; Table S6). This corresponds to an estimated delay of 28.43 months (95% CrI: 12.56 to 54.63 months). Notably, men with young‐onset LPA obtained a diagnosis 1.71 (95% CrI: 1.13 to 2.57) times longer than women from symptom onset (Figure 3; Table S4). This corresponds to an estimated delay of 20.94 months (95% CrI: 2.8 to 63.31 months).

### 4.4 Sensitivity analyses

All sensitivity analyses with alternative priors yielded results consistent with the main findings reported above (Tables S5–S8).

## 5 DISCUSSION

This study reveals important differences in diagnostic timelines across neurodegenerative conditions. Notably, patients with SD and bvFTD experienced longer diagnostic delays compared to those with AD, while patients with FTD‐MND received diagnoses sooner. Key sex differences emerged, as men with bvFTD experienced longer diagnostic delays than women with bvFTD. Subsequent analyses revealed these effects were isolated to younger onset (i.e., < 65 years). Similarly, in younger‐onset LPA, men were diagnosed later than women.

Limited research, likely due to small sample sizes, has examined variability in time to diagnosis from symptom onset between AD and FTD subtypes, with existing evidence suggesting no difference between AD and bvFTD.^25^ The present findings, which leveraged a much larger clinical sample (i.e., bvFTD cases *N* = 163 vs. *N* = 46^25^), suggest that those with bvFTD experience, on average, 1.23 years longer time to diagnosis compared to those with AD. These findings align with the clinical complexity of diagnosing bvFTD, given many of the symptoms overlap with those observed in psychiatric disorders and other dementias.^8^ Moreover, the distinctive nature of SD, which manifests subtly in everyday functioning, may impact patient and caregiver awareness of the extent of impairment,^9^ potentially contributing to delayed recognition and diagnosis. The earlier diagnosis observed in FTD‐MND compared to AD likely reflects the distinctive clinical presentation of concurrent motor symptoms alongside cognitive changes.^12^ The rapid progression of motor symptoms in FTD‐MND^12^ likely prompts patients to seek medical attention sooner, facilitating an earlier diagnosis. This inherent difference in symptom presentation underscores the need for sensitive preclinical biomarkers across the spectrum of neurodegenerative conditions to facilitate early identification before the marked progression in clinical manifestation.

Current diagnostic criteria may contribute to delays in formal diagnosis across neurodegenerative conditions. Analysis of our cohort revealed that of the 88 participants that did not meet the full diagnostic criteria on their first visit, the highest proportion within each diagnostic group was observed in individuals with bvFTD (AD = 7.2%, LPA = 12.7%, bvFTD = 25.2%, PNFA = 13.1%, SD = 4.8%, CBS = 21.7%, FTD‐MND = 11.8%, and PSP = 21.7%). Therefore, it is important to consider whether existing clinical criteria are excessively stringent for bvFTD. Moreover, limited awareness about FTD and specialist services among primary care physicians and radiologists may also contribute to diagnostic delays.

Our findings also reveal some important differences in diagnostic timelines between men and women with bvFTD that were not previously detected.^16^, ^25^, ^26^, ^27^, ^33^, ^35^, ^36^, ^37^ Specifically, men with bvFTD experience a longer time to diagnosis compared to women, with a delay of 1.97 years. Notably, these effects were isolated to the young‐onset group, and similar trends were detected in young‐onset LPA. These results may reflect sex‐based differences in health care–seeking behaviors or sociocultural factors influencing recognition of behavioral symptoms. For instance, in bvFTD, behavioral changes in men may be more likely to be attributed to personality traits rather than pathological processes, potentially delaying clinical evaluation. These findings highlight the importance of considering sex as a factor in diagnostic pathways for young‐onset bvFTD and LPA. Public health programs that emphasize the diverse early symptoms of neurodegenerative diseases may enhance early and accurate detection, facilitating timely access to health‐care services. Future research should explore the specific factors contributing to diagnosis delays, including patient, caregiver, and health‐care system influences to guide targeted strategies for timely diagnosis.

### 5.1 Strengths and limitations

The use of Bayesian regression allowed for several methodological advantages, including the incorporation of prior knowledge based on existing literature and expert opinion. This approach, combined with the relatively large sample size, helped guide the model toward plausible estimates while accounting for uncertainty, ensuring estimates remain realistic, robust, and precise. Moreover, sensitivity analyses with alternative priors yielded results consistent with the main findings, demonstrating the robustness of findings from prior specification. Another strength of the study includes specifying models that are appropriate for the data. Available evidence suggests a highly variable timeline for diagnosis among individuals with AD,^24^, ^25^, ^26^, ^27^, ^28^, ^29^, ^30^, ^31^, ^32^, ^33^ with average estimates ranging from 1 year^29^ to 5.5 years^31^ in duration. This variability may, in part, be accounted for by the non‐normal distribution of time to diagnosis from symptom onset, which is positively skewed, bounded by 0 (i.e., a diagnosis cannot be obtained prior to symptom onset) and consistently observed across neurodegenerative conditions (Figure 1). The use of a Bayesian regression model in this study with a gamma distribution and log link function provides a particularly suitable probabilistic framework, as it can effectively capture the skewed nature of diagnostic delay data, providing robust estimates of time to diagnosis and credible intervals that reflect the underlying distribution.

Limitations of this study include the reliance on reported symptom onset, which introduces potential recall bias, particularly given the retrospective nature of symptom reporting. The accuracy of self‐reported timelines may vary across conditions, with more insidious disorders (e.g., AD, SD, and bvFTD) presenting difficulties for patients and caregivers to date symptom onset. Furthermore, biomarkers of AD pathology (e.g., cerebrospinal fluid, positron emission tomography, or blood) were not used in this study, reflecting the diagnostic tools available at the time patients were assessed; however, all AD patients were typical of amnestic AD from a clinical perspective. There are also other key milestones in the diagnostic pathway including the time taken from initial symptom onset to the first consultation with a health‐care professional, as well as the time from that first consultation to receiving a first diagnosis and then, a diagnosis that meets clinical criteria. It is, therefore, not possible for us to determine whether the delays observed in this study are related to upstream effects, such as factors contributing to individuals delaying their first consultation with clinicians, or point to specific bottlenecks in the health system that hinder pathways to diagnosis. It is important to consider that the sample is drawn from a single dementia clinic in Sydney, Australia, whereby referral bias means the patients are young, overrepresented by FTD, underrepresented by typical late‐onset AD, in addition to dementia with Lewy bodies, vascular dementia, and mixed dementia. As such, further research will be needed to ensure whether our findings are generalizable to populations with different socio‐economic structures, as well as different dementia clinics or different health‐care systems.

## 6 CONCLUSION

In this study, patients with SD and bvFTD experienced longer diagnostic delays compared to those with AD, while patients with FTD‐MND received diagnoses sooner. Sex differences were evident for younger onset bvFTD and LPA, with men experiencing longer delays than women. These disparities may reflect syndrome‐specific clinical features, diagnostic complexity, and sociocultural factors influencing symptom recognition or health care–seeking behaviors. In bvFTD, longer delays may also stem from stringent diagnostic criteria. Public health efforts that promote awareness of the diverse early symptoms of neurodegenerative diseases could support early and accurate detection, facilitating timely access to health‐care services. These findings highlight the need to improve diagnostic pathways and emphasize the importance of developing preclinical biomarkers to enable timely identification prior to significant clinical progression.

## CONFLICT OF INTEREST STATEMENT

The authors declare no conflicts of interest. Author disclosures are available in the supporting information.

## CONSENT STATEMENT

All participants or their person responsible (i.e., spouse, family member, etc.) provided written informed consent in accordance with the Declaration of Helsinki. The South Eastern Sydney Local Health District, the University of New South Wales, and the University of Sydney human ethics committees approved the study (HREC 10/126, 2020/224 and 2020/408).

## Supporting information



Supplementary Figure 1. Prior and Posterior Predictive Check comparing observed diagnostic timelines (y) with model‐simulated data (y‐rep) for Diagnostic Group Analysis.

Supplementary Figure 2. Prior and Posterior Predictive Check comparing observed diagnostic timelines (y) with model‐simulated data (y‐rep) for Diagnostic Group by Onset Analysis.

Supplementary Figure 3. Prior and Posterior Predictive Check comparing observed diagnostic timelines (y) with model‐simulated data (y‐rep) for Diagnostic Group by Sex Analysis.

Supplementary Figure 4. Prior and Posterior Predictive Check comparing observed diagnostic timelines (y) with model‐simulated data (y‐rep) for Diagnostic Group by Sex for Young‐onset Analysis.

Supplementary Figure 5. Prior and Posterior Predictive Check comparing observed diagnostic timelines (y) with model‐simulated data (y‐rep) for Diagnostic Group by Sex for Late‐onset Analysis.

Supporting Information

Supporting Information
