# Supplementary figures and images for: A Bayesian analysis of diagnostic timelines across Alzheimer's disease, frontotemporal dementia, and other neurodegenerative conditions
Source: Alzheimers Dement (Amst). 2025 Sep 29;17(3):e70184. doi: 10.1002/dad2.70184 (PMC12477622; doi:10.1002/dad2.70184)

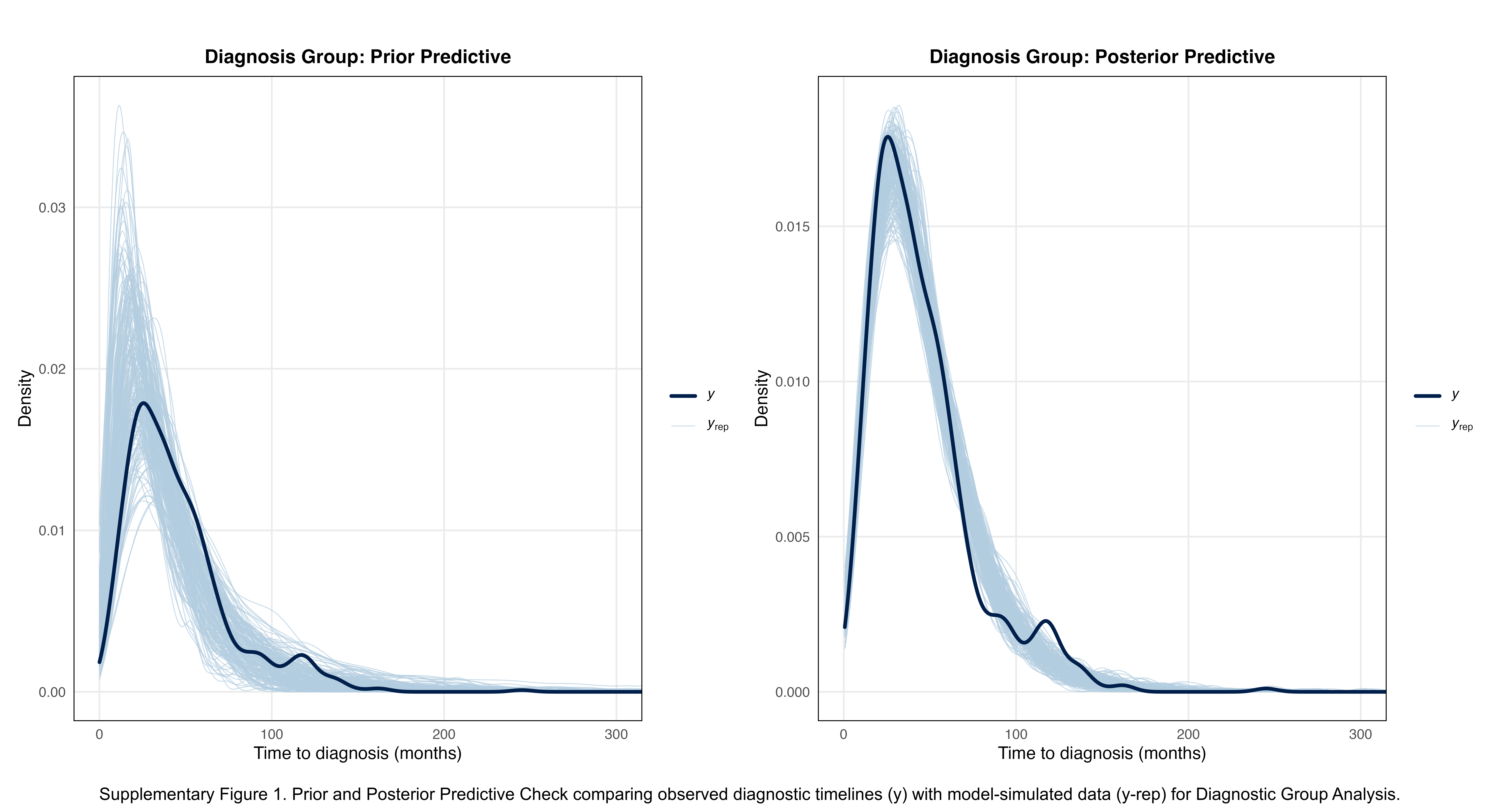

Supplement: Supplementary file 1 — Supplementary Figure 1. Prior and Posterior Predictive Check comparing observed diagnostic timelines (y) with model‐simulated data (y‐rep) for Diagnostic Group Analysis. [file DAD2-17-e70184-s001.tiff]

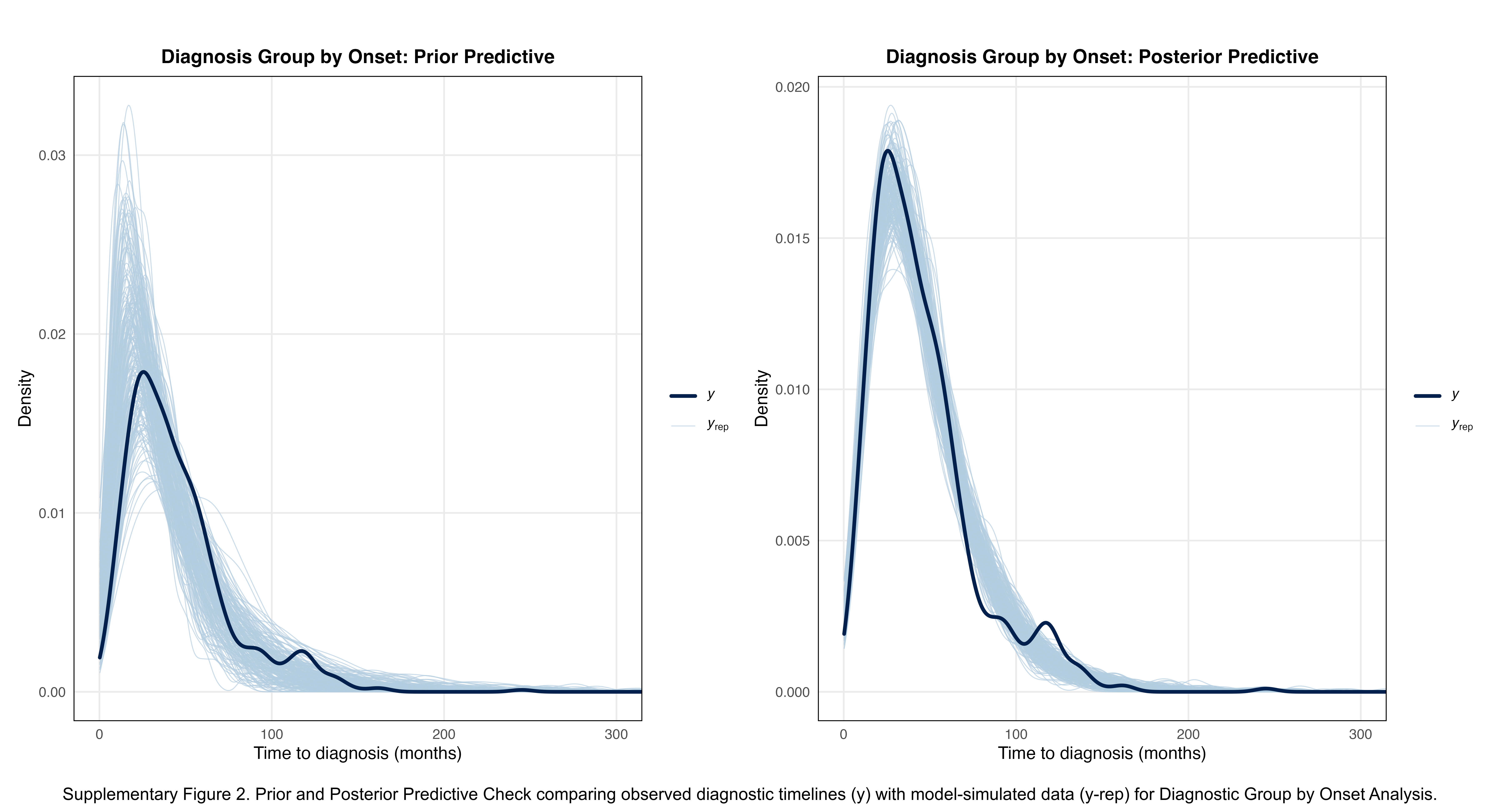

Supplement: Supplementary file 2 — Supplementary Figure 2. Prior and Posterior Predictive Check comparing observed diagnostic timelines (y) with model‐simulated data (y‐rep) for Diagnostic Group by Onset Analysis. [file DAD2-17-e70184-s004.tiff]

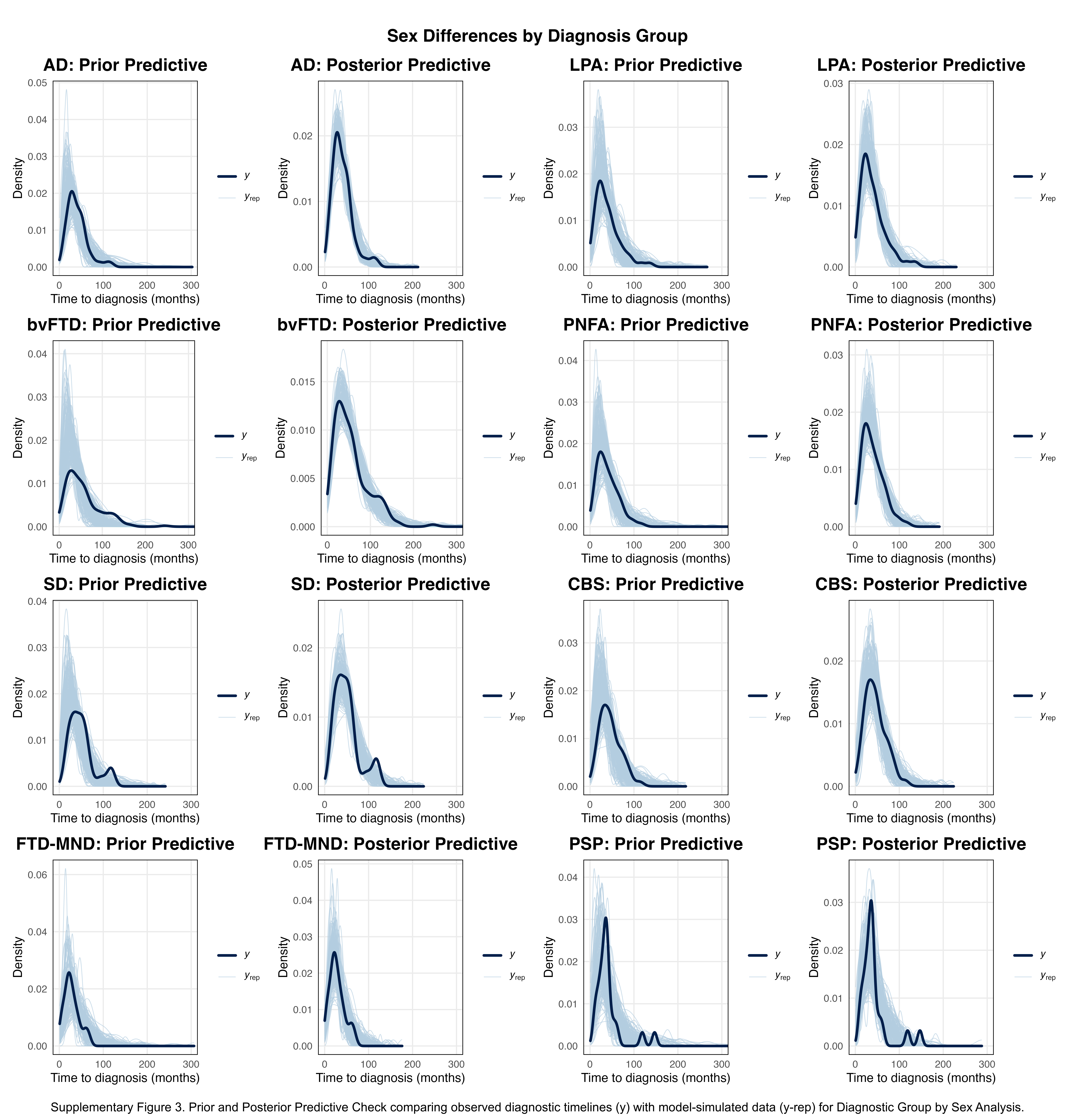

Supplement: Supplementary file 3 — Supplementary Figure 3. Prior and Posterior Predictive Check comparing observed diagnostic timelines (y) with model‐simulated data (y‐rep) for Diagnostic Group by Sex Analysis. [file DAD2-17-e70184-s003.tiff]

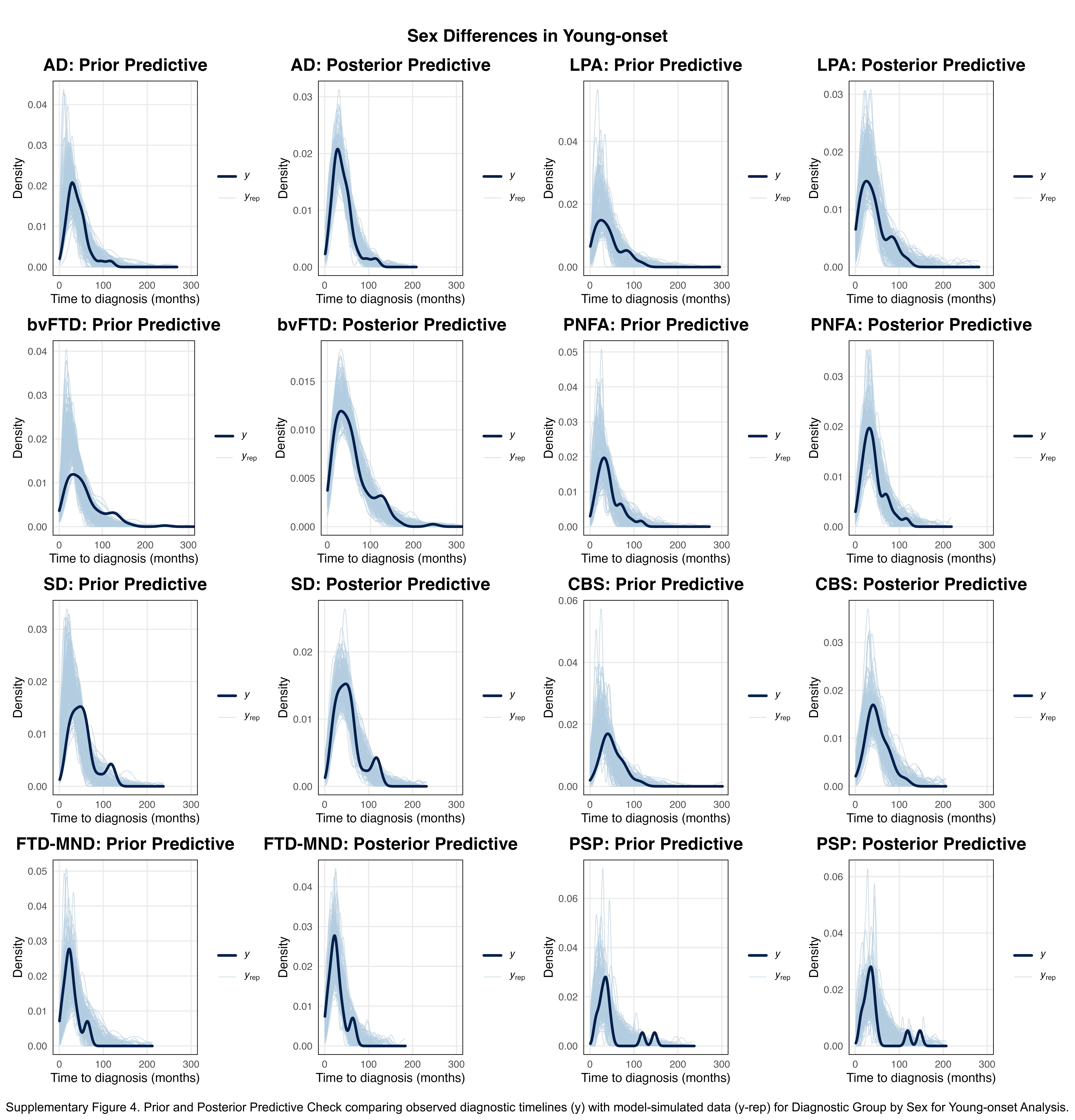

Supplement: Supplementary file 4 — Supplementary Figure 4. Prior and Posterior Predictive Check comparing observed diagnostic timelines (y) with model‐simulated data (y‐rep) for Diagnostic Group by Sex for Young‐onset Analysis. [file DAD2-17-e70184-s007.tiff]

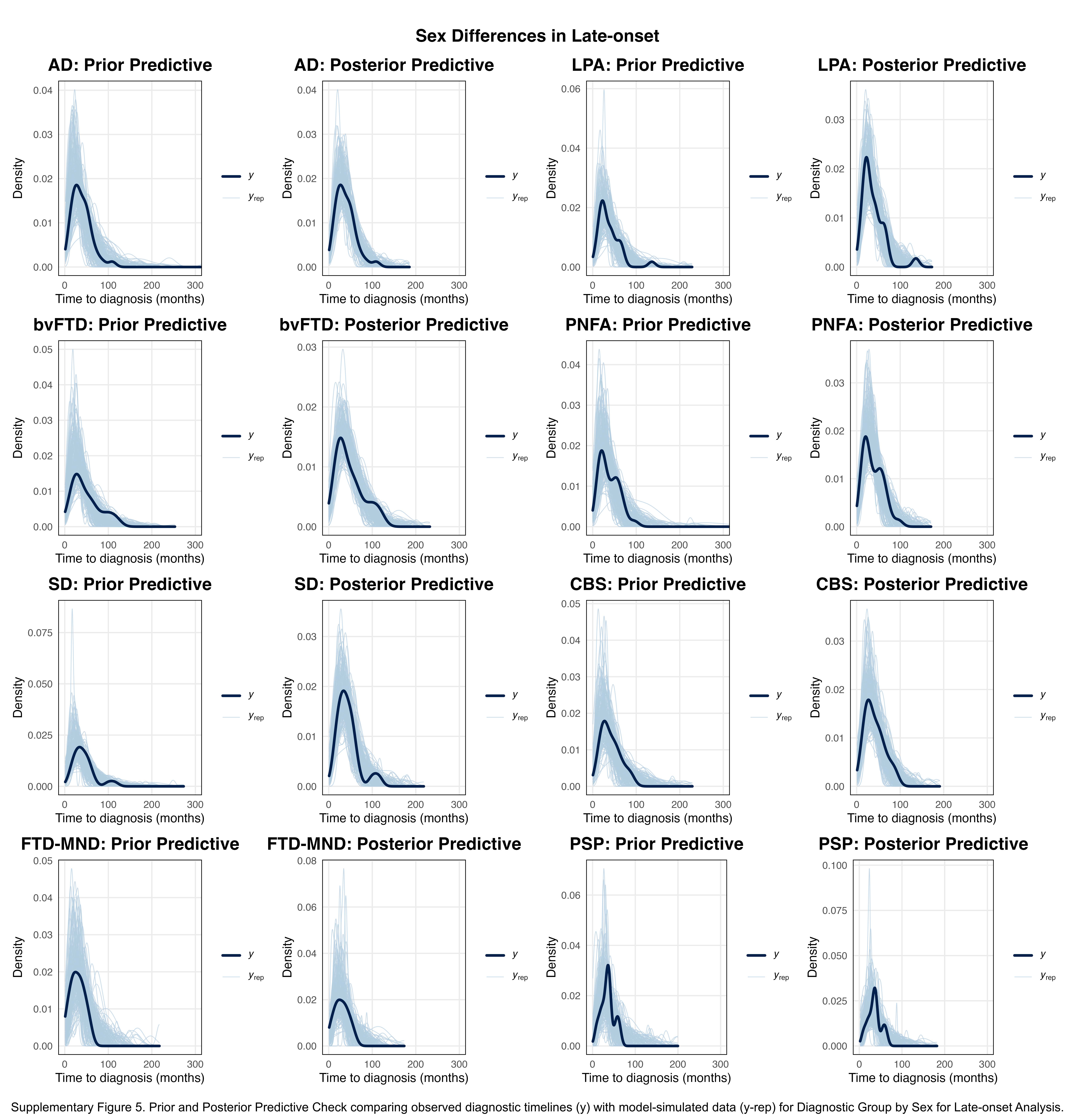

Supplement: Supplementary file 5 — Supplementary Figure 5. Prior and Posterior Predictive Check comparing observed diagnostic timelines (y) with model‐simulated data (y‐rep) for Diagnostic Group by Sex for Late‐onset Analysis. [file DAD2-17-e70184-s006.tiff]
