# Supplementary material for: A Bayesian analysis of diagnostic timelines across Alzheimer's disease, frontotemporal dementia, and other neurodegenerative conditions
Source: Alzheimers Dement (Amst). 2025 Sep 29;17(3):e70184. doi: 10.1002/dad2.70184 (PMC12477622; doi:10.1002/dad2.70184)
Supplement: Supplementary file 6 — Supporting Information [file DAD2-17-e70184-s005.docx]

Supplementary Table 1. Exponentiated Posterior Mean Estimates of Time to Diagnosis by Disease Group

| **Parameter** | **Estimate** | **Lower CrI** | **Upper CrI** | $\hat{\mathbf{R}}$ | **Bulk ESS** | **Tail ESS** |
| --- | --- | --- | --- | --- | --- | --- |
| Intercept (AD) | 40.21 | 36.31 | 44.62 | 1 | 42,940 | 39,902 |
| LPA | 0.99 | 0.82 | 1.19 | 1 | 60,291 | 45,720 |
| bvFTD | 1.37 | 1.19 | 1.57 | 1 | 48,901 | 46,398 |
| PNFA | 0.99 | 0.83 | 1.19 | 1 | 58,113 | 46,981 |
| SD | 1.24 | 1.05 | 1.46 | 1 | 56,237 | 45,293 |
| CBS | 1.13 | 0.92 | 1.38 | 1 | 63,469 | 45,990 |
| FTD-MND | 0.71 | 0.57 | 0.90 | 1 | 67,078 | 44,515 |
| PSP | 1.04 | 0.81 | 1.35 | 1 | 72,692 | 42,762 |

*Abbreviations: CrI, Credible Interval;* $\hat{R}$*, Potential Scale Reduction Factor; ESS, Effective Sample Size; AD, Alzheimer’s Disease; LPA, Logopenic Progressive Aphasia; bvFTD, Behavioural-Variant Frontotemporal Dementia; PNFA, Progressive Nonfluent Aphasia; SD, Semantic Dementia; CBS, Corticobasal Syndrome; FTD-MND, Frontotemporal Dementia with Motor Neuron Disease; PSP, Progressive Supranuclear Palsy.*

Supplementary Table 2. Exponentiated Posterior Mean Estimates of Time to Dementia Diagnosis by Disease Group and Onset

| **Parameter** | **Estimate** | **Lower CrI** | **Upper CrI** | $\hat{\mathbf{R}}$ | **Bulk ESS** | **Tail ESS** |
| --- | --- | --- | --- | --- | --- | --- |
| Intercept (Late-onset AD) | 39.48 | 33.78 | 46.33 | 1 | 12,830 | 21,842 |
| Young-onset AD | 1.03 | 0.85 | 1.26 | 1 | 17,135 | 29,397 |
| Late-onset LPA | 0.96 | 0.73 | 1.25 | 1 | 27,318 | 34,311 |
| Young-onset LPA | 1.06 | 0.81 | 1.39 | 1 | 27,294 | 36,986 |
| Late-onset bvFTD | 1.22 | 0.96 | 1.55 | 1 | 22,810 | 34,341 |
| Young-onset bvFTD | 1.45 | 1.20 | 1.75 | 1 | 16,378 | 28,172 |
| Late-onset PNFA | 0.96 | 0.74 | 1.25 | 1 | 27,108 | 36,984 |
| Young-onset PNFA | 1.06 | 0.82 | 1.37 | 1 | 26,418 | 36,717 |
| Late-onset SD | 1.09 | 0.83 | 1.45 | 1 | 29,101 | 34,756 |
| Young-onset SD | 1.33 | 1.07 | 1.65 | 1 | 20,108 | 32,147 |
| Late-onset CBS | 1.04 | 0.79 | 1.39 | 1 | 30,348 | 36,112 |
| Young-onset CBS | 1.25 | 0.95 | 1.67 | 1 | 29,578 | 35,576 |
| Late-onset FTD-MND | 0.77 | 0.54 | 1.13 | 1 | 43,310 | 40,189 |
| Young-onset FTD-MND | 0.72 | 0.54 | 0.96 | 1 | 30,535 | 37,373 |
| Late-onset PSP | 0.91 | 0.63 | 1.35 | 1 | 45,986 | 40,916 |
| Young-onset PSP | 1.17 | 0.84 | 1.67 | 1 | 39,660 | 39,828 |

*Abbreviations: CrI, Credible Interval;* $\hat{R}$*, Potential Scale Reduction Factor; ESS, Effective Sample Size; AD, Alzheimer’s Disease; LPA, Logopenic Progressive Aphasia; bvFTD, Behavioural-Variant Frontotemporal Dementia; PNFA, Progressive Nonfluent Aphasia; SD, Semantic Dementia; CBS, Corticobasal Syndrome; FTD-MND, Frontotemporal Dementia with Motor Neuron Disease; PSP, Progressive Supranuclear Palsy.*

Supplementary Table 3. Exponentiated Posterior Mean Estimates of Time to Dementia Diagnosis by Sex within Disease Group

| **Diagnosis Group** | **Parameter** | **Estimate** | **Lower CrI** | **Upper CrI** | $\hat{\mathbf{R}}$ | **Bulk ESS** | **Tail ESS** |
| --- | --- | --- | --- | --- | --- | --- | --- |
| AD | Intercept (Female) | 38.70 | 33.53 | 44.86 | 1 | 51,087 | 40,961 |
|  | Male | 1.06 | 0.87 | 1.29 | 1 | 49,900 | 40,183 |
| LPA | Intercept (Female) | 37.01 | 30.42 | 45.49 | 1 | 56,141 | 40,058 |
|  | Male | 1.15 | 0.84 | 1.57 | 1 | 55,424 | 40,969 |
| bvFTD | Intercept (Female) | 38.07 | 32.38 | 45.08 | 1 | 58,364 | 40,945 |
|  | Male | 1.62 | 1.32 | 1.98 | 1 | 56,717 | 42,135 |
| PNFA | Intercept (Female) | 39.07 | 32.16 | 47.91 | 1 | 54,627 | 40,584 |
|  | Male | 1.02 | 0.77 | 1.35 | 1 | 54,756 | 41,931 |
| SD | Intercept (Female) | 50.94 | 43.30 | 60.35 | 1 | 53,875 | 40,853 |
|  | Male | 0.92 | 0.73 | 1.16 | 1 | 52,009 | 40,744 |
| CBS | Intercept (Female) | 44.34 | 36.61 | 54.03 | 1 | 53,706 | 40,166 |
|  | Male | 0.99 | 0.73 | 1.36 | 1 | 51,705 | 41,762 |
| FTD-MND | Intercept (Female) | 29.84 | 20.86 | 43.85 | 1 | 48,522 | 37,365 |
|  | Male | 1.00 | 0.64 | 1.54 | 1 | 47,767 | 38,932 |
| PSP | Intercept (Female) | 40.93 | 30.46 | 55.65 | 1 | 58,692 | 41,602 |
|  | Male | 0.97 | 0.63 | 1.49 | 1 | 54,521 | 41,713 |

*Abbreviations: CrI, Credible Interval;* $\hat{R}$*, Potential Scale Reduction Factor; ESS, Effective Sample Size; AD, Alzheimer’s Disease; LPA, Logopenic Progressive Aphasia; bvFTD, Behavioural-Variant Frontotemporal Dementia; PNFA, Progressive Nonfluent Aphasia; SD, Semantic Dementia; CBS, Corticobasal Syndrome; FTD-MND, Frontotemporal Dementia with Motor Neuron Disease; PSP, Progressive Supranuclear Palsy.*

Supplementary Table 4. Exponentiated Posterior Mean Estimates of Time to Dementia Diagnosis by Sex within Disease Group and Onset

| **Diagnostic Group** | **Parameter** | **Estimate** | **Lower CrI** | **Upper CrI** | $\hat{\mathbf{R}}$ | **Bulk ESS** | **Tail ESS** |
| --- | --- | --- | --- | --- | --- | --- | --- |
| Young-onset AD | Intercept (Female) | 38.41 | 32.59 | 45.63 | 1 | 56,588 | 40,512 |
|  | Male | 1.11 | 0.88 | 1.39 | 1 | 56,727 | 42,793 |
| Young-onset LPA | Intercept (Female) | 29.68 | 22.27 | 40.26 | 1 | 52,832 | 40,327 |
|  | Male | 1.71 | 1.13 | 2.57 | 1 | 53,141 | 41,422 |
| Young-onset bvFTD | Intercept (Female) | 36.40 | 30.27 | 44.16 | 1 | 53,847 | 39,681 |
|  | Male | 1.78 | 1.41 | 2.24 | 1 | 53,010 | 42,050 |
| Young-onset PNFA | Intercept (Female) | 45.12 | 34.61 | 59.67 | 1 | 57,089 | 41,386 |
|  | Male | 0.82 | 0.56 | 1.20 | 1 | 54,636 | 40,594 |
| Young-onset SD | Intercept (Female) | 54.93 | 45.39 | 66.89 | 1 | 52,308 | 41,459 |
|  | Male | 0.86 | 0.66 | 1.13 | 1 | 52,238 | 40,860 |
| Young-onset CBS | Intercept (Female) | 44.22 | 34.39 | 57.32 | 1 | 50,500 | 40,739 |
|  | Male | 1.12 | 0.74 | 1.72 | 1 | 52,428 | 40,646 |
| Young-onset FTD-MND | Intercept (Female) | 32.34 | 21.94 | 48.95 | 1 | 52,794 | 38,405 |
|  | Male | 0.90 | 0.55 | 1.46 | 1 | 53,285 | 41,108 |
| Young-onset PSP | Intercept (Female) | 44.82 | 31.02 | 65.86 | 1 | 50,097 | 39,599 |
|  | Male | 0.90 | 0.53 | 1.54 | 1 | 48,685 | 40,163 |
| Late-onset AD | Intercept (Female) | 39.20 | 30.20 | 51.82 | 1 | 55,253 | 40,920 |
|  | Male | 0.96 | 0.67 | 1.36 | 1 | 53,734 | 41,929 |
| Late-onset LPA | Intercept (Female) | 41.93 | 33.54 | 52.91 | 1 | 53,523 | 40,368 |
|  | Male | 0.66 | 0.44 | 1.02 | 1 | 52,278 | 39,889 |
| Late-onset bvFTD | Intercept (Female) | 42.34 | 31.66 | 57.73 | 1 | 56,459 | 39,941 |
|  | Male | 1.13 | 0.78 | 1.63 | 1 | 54,394 | 40,575 |
| Late-onset PNFA | Intercept (Female) | 32.91 | 25.39 | 43.28 | 1 | 49,994 | 39,117 |
|  | Male | 1.29 | 0.88 | 1.89 | 1 | 47,761 | 39,935 |
| Late-onset SD | Intercept (Female) | 39.34 | 29.83 | 52.81 | 1 | 54,654 | 40,563 |
|  | Male | 1.11 | 0.74 | 1.68 | 1 | 52,692 | 40,583 |
| Late-onset CBS | Intercept (Female) | 42.22 | 32.48 | 55.77 | 1 | 59,515 | 42,459 |
|  | Male | 0.88 | 0.58 | 1.34 | 1 | 54,110 | 40,726 |
| Late-onset FTD-MND | Intercept (Female) | 27.07 | 14.49 | 53.56 | 1 | 56,191 | 39,775 |
|  | Male | 1.24 | 0.59 | 2.47 | 1 | 59,417 | 40,214 |
| Late-onset PSP | Intercept (Female) | 34.45 | 23.54 | 51.89 | 1 | 56,668 | 40,762 |
|  | Male | 1.08 | 0.61 | 1.92 | 1 | 55,038 | 41,179 |

*Abbreviations: CrI, Credible Interval;* $\hat{R}$*, Potential Scale Reduction Factor; ESS, Effective Sample Size; AD, Alzheimer’s Disease; LPA, Logopenic Progressive Aphasia; bvFTD, Behavioural-Variant Frontotemporal Dementia; PNFA, Progressive Nonfluent Aphasia; SD, Semantic Dementia; CBS, Corticobasal Syndrome; FTD-MND, Frontotemporal Dementia with Motor Neuron Disease; PSP, Progressive Supranuclear Palsy.*

Supplementary Table 5. Exponentiated Posterior Mean Estimates of Time to Diagnosis by Disease Group (Prior Sensitivity Analysis)

| **Parameter** | **Estimate** | **Lower CrI** | **Upper CrI** | **R-hat** | **Bulk ESS** | **Tail ESS** |
| --- | --- | --- | --- | --- | --- | --- |
| Intercept (AD) | 39.95 | 36.11 | 44.28 | 1 | 44,987 | 41,875 |
| LPA | 0.99 | 0.82 | 1.19 | 1 | 61,304 | 47,249 |
| bvFTD | 1.37 | 1.19 | 1.57 | 1 | 49,554 | 46,447 |
| PNFA | 0.99 | 0.83 | 1.19 | 1 | 59,412 | 47,259 |
| SD | 1.24 | 1.06 | 1.46 | 1 | 56,614 | 47,426 |
| CBS | 1.13 | 0.93 | 1.38 | 1 | 62,513 | 46,166 |
| FTD-MND | 0.71 | 0.57 | 0.89 | 1 | 69,622 | 46,813 |
| PSP | 1.04 | 0.80 | 1.35 | 1 | 74,666 | 44,203 |

*Abbreviations: CrI, Credible Interval;* $\hat{R}$*, Potential Scale Reduction Factor; ESS, Effective Sample Size; AD, Alzheimer’s Disease; LPA, Logopenic Progressive Aphasia; bvFTD, Behavioural-Variant Frontotemporal Dementia; PNFA, Progressive Nonfluent Aphasia; SD, Semantic Dementia; CBS, Corticobasal Syndrome; FTD-MND, Frontotemporal Dementia with Motor Neuron Disease; PSP, Progressive Supranuclear Palsy.*

Supplementary Table 6. Exponentiated Posterior Mean Estimates of Time to Dementia Diagnosis by Disease Group and Onset (Prior Sensitivity Analysis)

| **Parameter** | **Estimate** | **Lower CrI** | **Upper CrI** | **Rhat** | **Bulk ESS** | **Tail ESS** |
| --- | --- | --- | --- | --- | --- | --- |
| Intercept (Late-onset AD) | 39.29 | 33.66 | 46.00 | 1 | 13,653 | 23,871 |
| Young-onset AD | 1.03 | 0.85 | 1.26 | 1 | 19,146 | 32,604 |
| Late-onset LPA | 0.95 | 0.73 | 1.25 | 1 | 30,036 | 36,840 |
| Young-onset LPA | 1.06 | 0.81 | 1.39 | 1 | 29,628 | 35,979 |
| Late-onset bvFTD | 1.22 | 0.96 | 1.55 | 1 | 25,485 | 37,947 |
| Young-onset bvFTD | 1.45 | 1.20 | 1.74 | 1 | 17,211 | 30,225 |
| Late-onset PNFA | 0.96 | 0.74 | 1.25 | 1 | 29,581 | 36,799 |
| Young-onset PNFA | 1.05 | 0.82 | 1.36 | 1 | 28,881 | 37,765 |
| Late-onset SD | 1.09 | 0.83 | 1.45 | 1 | 31,466 | 36,846 |
| Young-onset SD | 1.33 | 1.07 | 1.65 | 1 | 21,436 | 34,584 |
| Late-onset CBS | 1.04 | 0.79 | 1.38 | 1 | 33,134 | 39,028 |
| Young-onset CBS | 1.25 | 0.95 | 1.66 | 1 | 31,922 | 37,378 |
| Late-onset FTD-MND | 0.77 | 0.54 | 1.13 | 1 | 49,394 | 41,458 |
| Young-onset FTD-MND | 0.72 | 0.54 | 0.96 | 1 | 32,440 | 38,563 |
| Late-onset PSP | 0.91 | 0.63 | 1.35 | 1 | 47,451 | 41,072 |
| Young-onset PSP | 1.17 | 0.84 | 1.66 | 1 | 42,798 | 39,104 |

*Abbreviations: CrI, Credible Interval;* $\hat{R}$*, Potential Scale Reduction Factor; ESS, Effective Sample Size; AD, Alzheimer’s Disease; LPA, Logopenic Progressive Aphasia; bvFTD, Behavioural-Variant Frontotemporal Dementia; PNFA, Progressive Nonfluent Aphasia; SD, Semantic Dementia; CBS, Corticobasal Syndrome; FTD-MND, Frontotemporal Dementia with Motor Neuron Disease; PSP, Progressive Supranuclear Palsy.*

Supplementary Table 7. Exponentiated Posterior Mean Estimates of Time to Dementia Diagnosis by Sex within Disease Group (Prior Sensitivity Analysis)

| **Diagnosis Group** | **Parameter** | **Estimate** | **Lower CrI** | **Upper CrI** | **R-hat** | **Bulk ESS** | **Tail ESS** |
| --- | --- | --- | --- | --- | --- | --- | --- |
| AD | Intercept (Female) | 37.79 | 32.84 | 43.73 | 1 | 53,007 | 41,314 |
|  | Male | 1.06 | 0.87 | 1.28 | 1 | 53,016 | 41,212 |
| LPA | Intercept (Female) | 34.85 | 28.68 | 42.61 | 1 | 52,620 | 40,388 |
|  | Male | 1.15 | 0.84 | 1.57 | 1 | 52,507 | 39,484 |
| bvFTD | Intercept (Female) | 37.22 | 31.65 | 44.01 | 1 | 54,915 | 39,560 |
|  | Male | 1.62 | 1.32 | 1.98 | 1 | 53,374 | 41,131 |
| PNFA | Intercept (Female) | 37.24 | 30.73 | 45.56 | 1 | 53,907 | 41,751 |
|  | Male | 1.02 | 0.77 | 1.35 | 1 | 54,572 | 41,570 |
| SD | Intercept (Female) | 49.24 | 41.86 | 58.01 | 1 | 52,135 | 42,710 |
|  | Male | 0.93 | 0.74 | 1.16 | 1 | 56,487 | 42,985 |
| CBS | Intercept (Female) | 41.87 | 34.72 | 50.86 | 1 | 52,844 | 40,329 |
|  | Male | 0.99 | 0.73 | 1.34 | 1 | 52,082 | 40,389 |
| FTD-MND | Intercept (Female) | 27.25 | 19.30 | 39.22 | 1 | 51,870 | 38,054 |
|  | Male | 1.01 | 0.66 | 1.52 | 1 | 52,106 | 39,110 |
| PSP | Intercept (Female) | 36.82 | 27.63 | 49.51 | 1 | 48,259 | 41,910 |
|  | Male | 0.97 | 0.64 | 1.47 | 1 | 47,642 | 38,836 |

*Abbreviations: CrI, Credible Interval;* $\hat{R}$*, Potential Scale Reduction Factor; ESS, Effective Sample Size; AD, Alzheimer’s Disease; LPA, Logopenic Progressive Aphasia; bvFTD, Behavioural-Variant Frontotemporal Dementia; PNFA, Progressive Nonfluent Aphasia; SD, Semantic Dementia; CBS, Corticobasal Syndrome; FTD-MND, Frontotemporal Dementia with Motor Neuron Disease; PSP, Progressive Supranuclear Palsy.*

Supplementary Table 8. Exponentiated Posterior Mean Estimates of Time to Dementia Diagnosis by Sex within Disease Group and Onset (Prior Sensitivity Analysis)

| **Diagnostic Group** | **Parameter** | **Estimate** | **Lower CrI** | **Upper CrI** | **Rhat** | **Bulk ESS** | **Tail ESS** |
| --- | --- | --- | --- | --- | --- | --- | --- |
| Young-onset AD | Intercept (Female) | 37.13 | 31.55 | 43.97 | 1 | 55,157 | 38,369 |
|  | Male | 1.11 | 0.88 | 1.39 | 1 | 55,372 | 40,239 |
| Young-onset LPA | Intercept (Female) | 26.75 | 20.16 | 35.98 | 1 | 50,522 | 39,361 |
|  | Male | 1.72 | 1.15 | 2.56 | 1 | 53,398 | 40,715 |
| Young-onset bvFTD | Intercept (Female) | 35.32 | 29.33 | 42.84 | 1 | 57,011 | 42,744 |
|  | Male | 1.78 | 1.42 | 2.24 | 1 | 55,776 | 41,471 |
| Young-onset PNFA | Intercept (Female) | 41.52 | 32.02 | 54.45 | 1 | 50,285 | 41,001 |
|  | Male | 0.82 | 0.57 | 1.18 | 1 | 49,005 | 39,871 |
| Young-onset SD | Intercept (Female) | 52.47 | 43.37 | 63.81 | 1 | 51,094 | 39,867 |
|  | Male | 0.86 | 0.66 | 1.13 | 1 | 50,042 | 40,724 |
| Young-onset CBS | Intercept (Female) | 40.09 | 31.43 | 51.37 | 1 | 48,434 | 41,256 |
|  | Male | 1.12 | 0.76 | 1.68 | 1 | 47,436 | 38,792 |
| Young-onset FTD-MND | Intercept (Female) | 28.80 | 19.93 | 42.80 | 1 | 54,261 | 39,798 |
|  | Male | 0.90 | 0.56 | 1.43 | 1 | 53,774 | 39,379 |
| Young-onset PSP | Intercept (Female) | 38.41 | 26.82 | 55.60 | 1 | 48,820 | 40,682 |
|  | Male | 0.90 | 0.54 | 1.50 | 1 | 50,328 | 40,057 |
| Late-onset AD | Intercept (Female) | 36.50 | 28.24 | 47.71 | 1 | 54,798 | 40,447 |
|  | Male | 0.96 | 0.68 | 1.34 | 1 | 54,794 | 40,536 |
| Late-onset LPA | Intercept (Female) | 38.32 | 30.79 | 47.93 | 1 | 52,410 | 40,609 |
|  | Male | 0.66 | 0.44 | 1.00 | 1 | 55,501 | 39,790 |
| Late-onset bvFTD | Intercept (Female) | 39.26 | 29.52 | 52.91 | 1 | 48,846 | 39,013 |
|  | Male | 1.13 | 0.79 | 1.61 | 1 | 50,444 | 40,221 |
| Late-onset PNFA | Intercept (Female) | 30.19 | 23.47 | 39.27 | 1 | 49,005 | 40,399 |
|  | Male | 1.30 | 0.90 | 1.88 | 1 | 50,817 | 42,141 |
| Late-onset SD | Intercept (Female) | 35.68 | 27.33 | 47.15 | 1 | 52,904 | 40,637 |
|  | Male | 1.12 | 0.75 | 1.65 | 1 | 53,826 | 41,427 |
| Late-onset CBS | Intercept (Female) | 38.22 | 29.57 | 49.90 | 1 | 51,822 | 40,433 |
|  | Male | 0.88 | 0.58 | 1.32 | 1 | 46,877 | 38,182 |
| Late-onset FTD-MND | Intercept (Female) | 22.15 | 12.25 | 42.28 | 1 | 50,758 | 38,229 |
|  | Male | 1.27 | 0.63 | 2.44 | 1 | 51,926 | 38,480 |
| Late-onset PSP | Intercept (Female) | 28.86 | 20.05 | 42.35 | 1 | 51,101 | 38,387 |
|  | Male | 1.09 | 0.63 | 1.88 | 1 | 50,308 | 40,792 |

*Abbreviations: CrI, Credible Interval;* $\hat{R}$*, Potential Scale Reduction Factor; ESS, Effective Sample Size; AD, Alzheimer’s Disease; LPA, Logopenic Progressive Aphasia; bvFTD, Behavioural-Variant Frontotemporal Dementia; PNFA, Progressive Nonfluent Aphasia; SD, Semantic Dementia; CBS, Corticobasal Syndrome; FTD-MND, Frontotemporal Dementia with Motor Neuron Disease; PSP, Progressive Supranuclear Palsy.*
